# Our Limbless Sailors and Soldiers: An Appeal

**Published:** 1915-10-02

**Authors:** Francis Lloyd, C. H. Kenderdine

**Affiliations:** Chairman.; Honorary Treasurer. St. Stephen's House, Westminster, S.W.


### THE EDITOR'S LETTER-BOX.

### Our Limbless Sailors and Soldiers: An Appeal.

*To the Editor of* The Hospital.

Sir,—The nation owes a special debt of gratitude to those gallant men who have lost their limbs in the war. On their behalf we make an earnest appeal for additional funds to extend the work at Queen Mary's Convalescent Hospitals, Roehampton, where these sufferers (officers and men) are provided at the expense of the State with their artificial appliances and taught how to use them. The hospitals are officially recognised by the Directors-General of the Navy and Army Medical Services and the Lords Commissioners of the Royal Hospital, Chelsea.

Over eight hundred patients are now awaiting admission, among them many men from our Overseas Dominions, and the numbers increase daily. To meet this difficult situation a large outlay has been incurred in the erection and equipment of new wards, which will shortly be opened.

The work at Roehampton can best be judged by the results achieved and by the gratitude of these brave men on realising that, with the aid of the wonderful artificial limbs of recent invention, they will be able to obtain employment and make a fresh start in life.

With a view to their future employment and also to provide useful occupation for the men while in hospital, workshops fitted with model motor chassis, electrical appliances, lathes, etc., are being organised, with competent instructors. A man who has lost his right hand will be taught to write with the left, and classes for other industries will be arranged. An employment bureau working in conjunction with existing societies and employers of labour has also been established, and already a number of men have been placed in good situations. From the numerous offers received, it is hoped through this medium to find suitable employment for every man on his leaving the hospital.

To maintain and extend this national work a large additional sum of money is required, and we look with confidence to a ready response to our appeal from public and private sources, notwithstanding the urgency of other claims. Donations sent to the honorary treasurer at St. Stephen's House, Westminster, will be gratefully acknowledged.—Yours obediently.

Francis Lloyd (Major-General), Chairman.
C. H. Kenderdine, Honorary Treasurer.

St. Stephen's House, Westminster, S.W.,
September 25, 1915.

